# Perfluorobutanoic Acid (PFBA) Induces a Non-Enzymatic Oxidative Stress Response in Soybean (*Glycine max* L. Merr.)

**DOI:** 10.3390/ijms23179934

**Published:** 2022-09-01

**Authors:** Eguono W. Omagamre, Yeganeh Mansourian, Diamond Liles, Tigist Tolosa, Simon A. Zebelo, Joseph S. Pitula

**Affiliations:** 1 Department of Natural Sciences, University of Maryland Eastern Shore, Princess Anne, MD 21853, USA; 2 Department of Agricultural and Food Sciences, University of Maryland Eastern Shore, Princess Anne, MD 21853, USA

**Keywords:** *Glycine max*, PFAS, PFBA, phytotoxicity, circadian rhythm, cryptochrome, glycine betaine, hormesis, RNA-seq, circadian clock genes

## Abstract

Short-chain perfluoroalkyl substances (PFAS) are generally considered to be of less environmental concern than long-chain analogues due to their comparatively shorter half-lives in biological systems. Perfluorobutanoic acid (PFBA) is a short-chain PFAS with the most root–shoot transfer factor of all PFAS. We investigated the impact of extended exposure of soybean plants to irrigation water containing environmentally relevant (100 pg–100 ng/L) to high (100 µg–1 mg/L) concentrations of PFBA using phenotypical observation, biochemical characterization, and transcriptomic analysis. The results showed a non-monotonous developmental response from the plants, with maximum stimulation and inhibition at 100 ng/L and 1 mg/L, respectively. Higher reactive oxygen species and low levels of superoxide dismutase (SOD) and catalase (CAT) activity were observed in all treatment groups. However transcriptomic analysis did not demonstrate differential expression of SOD and CAT coding genes, whereas non-enzymatic response genes and pathways were enriched in both groups (100 ng/L and 1 mg/L) with glycine betaine dehydrogenase showing the highest expression. About 18% of similarly downregulated genes in both groups are involved in the ethylene signaling pathway. The circadian rhythm pathway was the only differentially regulated pathway between both groups. We conclude that, similar to long chain PFAS, PFBA induced stress in soybean plants and that the observed hormetic stimulation at 100 ng/L represents an overcompensation response, via the circadian rhythm pathway, to the induced stress.

## 1. Introduction

Per and polyfluoroalkyl substances (PFAS) are a class of chemical contaminants of emerging concern. Recent reviews of available soil data worldwide suggest that PFAS-free soil is rare [1,2,3,4,5]. Major sources of soil and plant PFAS accumulation are via irrigation with contaminated well water, surface runoff, and wastewater effluent [1,2,6].

When plants transport water and nutrients from soils, xenobiotics can be co-transported into the plant roots [7], and a positive correlation between PFAS soil/water concentration and plant uptake and transport has been demonstrated [8,9]. The comparatively small bond diameter of PFAS molecules (1.3 Å) has led researchers to suggest aquaporins (2–3 Å pore diameter) as a possible channel of PFAS transport into plants [10]. Physicochemical properties also play an important role in bioavailability, with chain-length and functional groups influencing bioaccumulation [3,11]. For example, twice as much of the short-chain PFAS, perfluorobutanoic acid (PFBA), was observed to accumulate in *Bromus diandrus* grass as compared to perfluorobutane sulfonic acid (PFBS) [9]. The soil-to-root transport of short-chain PFAS is considered high due to their low adsorption to soil molecules compared to long-chain PFAS. The comparatively high-water solubility and low lipophilicity of the short-chain PFAS also limit their interactions with proteins in plant root systems, facilitating transport to the shoots and aerial parts of plants [8,12,13].

Studies on the phytotoxic impacts of PFAS are emerging [1,14,15]. PFAS have been shown to modulate plant biomass accumulation, leaf chlorophyll content, and other enzymatic activities [16,17,18]. A study by Li et al. [19] observed an upregulation of non-enzymatic antioxidants in response to PFOS exposure in lettuce. Similarly, PFOA and PFOS perturbed the Tricarboxylic acid (TCA) cycle and pyruvate metabolism in lettuce leaves [20]. Exposure of *Arabidopsis thaliana* to 100 µM PFOA upregulated 11 genes that are normally induced by iron deficiency [21]. Short-chain PFAS have also been suggested to cause similar phytotoxic effects though the experimental exposure concentrations used were higher than environmentally relevant concentrations, thus making extrapolation of phytotoxicity data to field conditions difficult [1,22].

The goal of this study was to determine what exposure levels of PFBA (the PFAS known to show the most aerial transport in plants) produced toxic impacts in soybean plants, and whether any toxic effects are induced at environmentally relevant concentrations. Moreover, in our previous study [23], we observed that beet armyworm larvae had significantly higher weight gain when fed on leaves from soybean plants grown with a low concentration of PFBA (10–100 ng/L) as compared to higher concentrations (100 µg–1 mg/L). As PFBA has been shown to also induce a non-linear impact in the rotifer *Brachionus calyciflorus* [24] we hypothesized that PFBA could modulate key developmental indices in exposed plants in a non-monotonic dose-response manner. Using a combination of phenotypical observation, biochemical characterization, and transcriptomic analysis we observed a distinct stress response in all treatment groups, despite a hormetic response at 100 ng/L.

## 2. Results

### 2.1. Effect of PFBA Concentration on Plant Development

#### 2.1.1. Plant Growth

**Figure 1:**
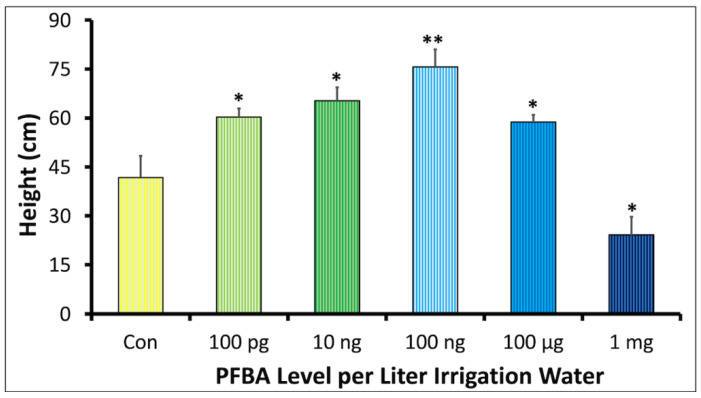
Dose–response relationship between the concentration of PFBA in irrigation water and plant height of soybean plants grown for 5 weeks. All plants received 1.4 L of irrigation water. The control group (Con) was irrigated with Nanopure water (Barnstead, Thermofisher) (*n* = 3). Plant height data were fitted to the Brain–Cousens 4 parameter model. The *f* parameter suggests insignificant hormesis at *p* < 0.05 (−156 < *f* < 184). The * and ** symbols indicate significant difference between the treatment and control at *p* < 0.05 and *p* < 0.01, respectively.

Unlike studies where the PFBA exposure commenced with already germinated seedlings [1], our study measured cumulative exposure impacts from seed germination through seedling development, until the plants had grown for 5 weeks post-planting. Plant height data for soybean plants exposed to varying concentrations of PFBA are shown in Figure 1. The control plants attained a mean height of 41.8 ± 6.6 cm. The 1 mg/L treatment showed a significantly depressed growth of 24.9 ± 5.5 cm when compared with the controls. By contrast, the height at the 100 pg/L, 10 ng/L, 100 ng/L and 100 µg/L treatments were significantly higher than the controls. The plant height data fitted a Brain–Cousens 4-parameter non-monotonic dose-response model for hormesis. However, the bounds of hormesis at *p* < 0.05 were observed to span zero i.e., −156 < *f* < 184, suggesting that the observed hormesis was not significant (Supplemental S1—Table S1).

#### 2.1.2. Leaf Morphology and Chlorophyll Content

**Figure 2:**
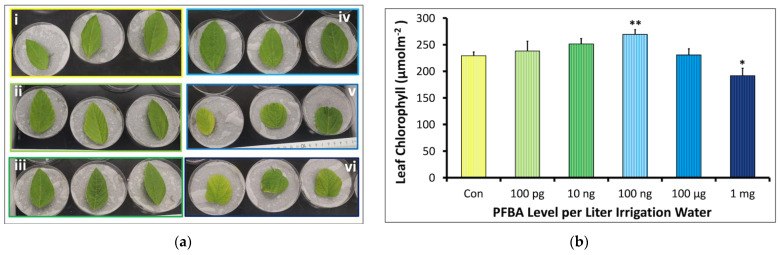
Dose–response relationship between the concentration of PFBA in irrigation water in leaves of soybean plants after 5 weeks of planting and exposure (*n* = 3). (**a**) shows leaf morphology, (**i**) control, (**ii**) 100 pg/L, (**iii**) 10 ng/L, (**iv**) 100 ng/L, (**v**) 100 μg/L, and (**vi**) 1 mg/L groups. Leaves appeared turgid, heavier, and brittle at higher PFBA treatments. About 67% of the leaves in the 100 μg/L replicates had shrunken edges but green, while the remaining appeared smallish and yellowish (**b**) chlorophyll *a* content. The * and ** symbols indicate significant difference between the treatment and control at *p* < 0.05 and *p* < 0.01, respectively.

Leaf morphologies also were influenced by the varying PFBA treatments, with shrinking structures in the 100 µg, and the 1 mg groups, suggesting toxicity (Figure 2a). A physical inspection suggested a linear response in increasing leaf vein, turgidity, and surface roughness in the higher treatment groups (i.e., 100 ng, 100 µg, and the 1 mg groups) when compared with the controls. As shown in Figure 2b, plants treated with 1 mg/L PFBA had significantly lower levels of chlorophyll *a* (Chl) relative to the control (192 ± 14 µmolm^−2^ Chl compared to 229 ± 7; *p* = 0.026). All other treatment groups showed higher Chl concentrations, with the 100 ng/L treatment being statistically significant (231 ± 12 µmolm^−2^ Chl; *p* = 0.006). These observations were consistent with the ribosomal rRNA analysis, which suggested chloroplast ribosomal expression [25] followed a 100 ng/L > controls > 1 mg/L pattern (Supplemental S1—Figure S1). The Chl data were also fitted with the Brain–Cousens model (Supplemental S1—Figure S2 and Table S2). An estimated degree of leaf Chl stimulation at doses close to zero (*f parameter*) was 20. The bounds of hormesis of pigmentation at 95% Confidence Interval indicates *f* > 0 (i.e., 5 < *f* < 35), which confirms significant hormesis. The value of *f* suggests low stimulation which is supported by a 22% maximum stimulation of controls. Stimulation levels that fall outside the generalized 30–60% stimulation range have been reported in other studies [26,27]. A positive correlation with an r^2^ of 0.64 was observed between the leaf Chl and the plant height (Supplemental S1—Figure S3).

#### 2.1.3. Effect of PFBA on Flavonoid Content

**Figure 3:**
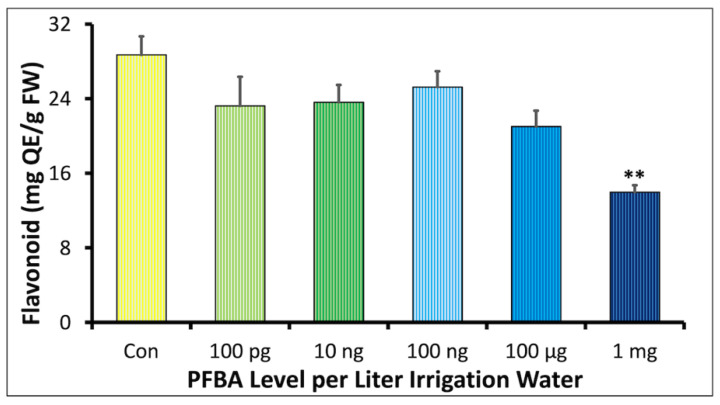
Dose–response relationship between the concentration of PFBA in irrigation water and leaf flavonoid content in soybean plants grown for 5 weeks. The ** symbol indicates significant difference between the treatment and control at *p* < 0.01, respectively.

The flavonoid (Fla) content observed was expressed as mg quercetin equivalent (QE) per gram fresh weight (FW) (*n* = 3). The QE in the treatment groups ranged from 13.95 to 25.24 mg QE/g FW (Figure 3). These concentrations were slightly lower than those observed for the controls, i.e., 28.70 ± 1.96 QE/g FW. The 100 ng/L group showed the most Fla content of all the treatment groups, i.e., 25.24 ± 1.68 QE/g FW while the 1 mg/L group showed the lowest level, i.e., 13.96 ± 0.77 QE/g FW. The decrease in the 1 mg/L group compared to the controls was significant.

### 2.2. Effect of PFBA on Stress Molecules, Antioxidant Stress Enzymes, and Metabolites

#### 2.2.1. Total Reactive Oxygen Species (ROS) and Hydrogen Peroxide (H_2_O_2_)

**Figure 4:**
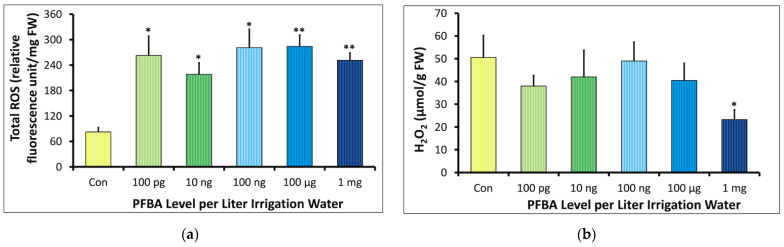
Dose–response relationship between the concentration of PFBA in irrigation water and reactive oxygen species in leaves of soybean plant grown for 5 weeks. The * and ** symbols indicate significant difference between the treatment and control at *p* < 0.05 and *p* < 0.01, respectively (*n* = 3). (**a**) shows the total reactive oxygen (ROS) concentration across all groups, with higher levels observed in the PFBA treatments compared to the controls, (**b**) shows the hydrogen peroxide (H_2_O_2_) levels did not increase as observed for systems with increased ROS.

The ROS levels were higher in all treatment groups compared to the controls (Figure 4a). The ROS levels in the treatment groups ranged from 218.01 to 284.03 rel. fluorescence unit/mg FW compared to the 82.24 rel. fluorescence unit/mg FW in the controls. The difference in ROS levels in all treatment groups as compared to the controls was significant.

The H_2_O_2_ content did not increase in the treatments as would be expected for systems showing elevated ROS levels (Figure 4b). The H_2_O_2_ concentration decreased from 38.02 ± 4.61 µmol/g FW in the 100 pg/L group to 23.23 ± 4.33 µmol/g FW in the 1 mg/L group. The controls showed higher H_2_O_2_ levels than all groups with a value of 50.57 ± 9.60 µmol/g FW, which was statistically significant when compared to the 1 mg/L group.

#### 2.2.2. Superoxide Dismutase (SOD) and Catalase (CAT)

**Figure 5:**
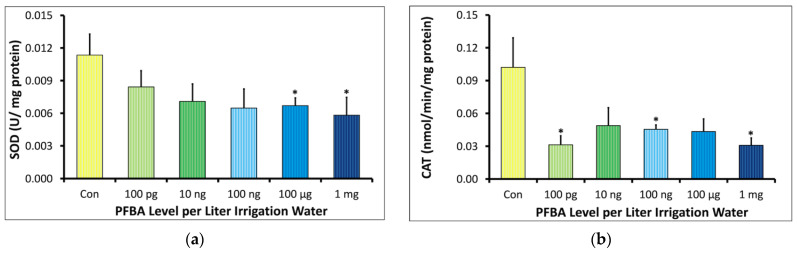
Dose–response relationship between concentration of PFBA in irrigation water and enzymes involved in detoxification of reactive oxygen species in leaves of soybean plant grown for 5 weeks. The * symbols indicate significant difference between the treatment and control at *p* < 0.05 and *p* < 0.01, respectively (*n* = 3). (**a**) shows the superoxide dismutase (SOD) levels which decreased significantly in the 100 ng/L and 1 mg/L groups. (**b**) shows decreased catalase (CAT) levels in all treatment groups compared to the control.

For SOD activity, an almost linear decline in response was observed with an increasing concentration of PFBA (Figure 5a). The controls had the highest SOD activity of 0.011 ± 0.002 U/mg protein, while the treatment groups ranged from 0.006 to 0.008 U/mg protein. The decrease in SOD activity was significant in the 100 µg/L and 1 mg/L groups. The CAT levels in controls were observed to be double of the maximum levels observed in the treatment groups (Figure 5b). CAT activity in the treatments ranged from 0.031 to 0.049 nmolmin^−1^mg^−1^ protein as compared to the 0.10 nmol min^−1^mg^−1^ protein observed in the controls.

### 2.3. General Transcriptome and Differential Gene Expression Profile

The physiological response of plants to PFBA indicated that plants experienced oxidative stress at all treatments, despite the non-monotonous developmental patterns observed. To gain insight into the basis for these physiological responses, transcription libraries from the control, 100 ng/L, and 1 mg/L groups (*n* = 3) were constructed, sequenced, and trimmed, generating a mean number of reads of 22.5M, 21.8M, and 21.2M, respectively. After mapping the reads to the soybean genome, an average of 50,000 nonzero gene transcripts were obtained for each of the three groups (Supplemental S2). The transcripts of the replicates of each of the treatment groups clustered together (Supplemental S1—Figure S4).

**Figure 6:**
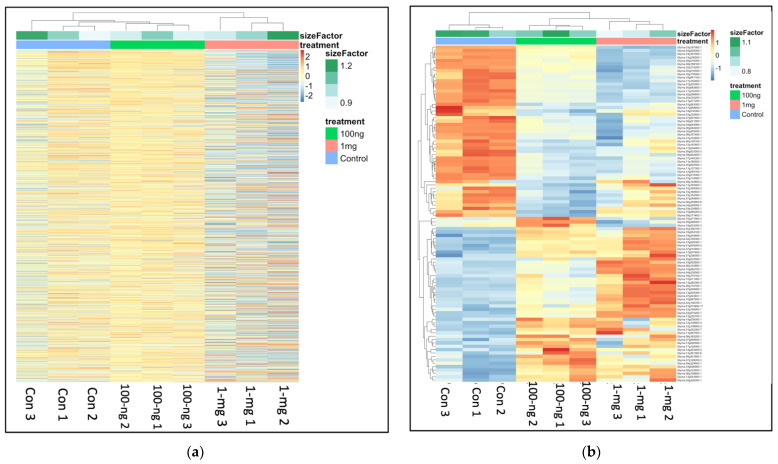
Clustering profile of (**a**) top 1000 expressed genes of the control, 100 ng/L and 1 mg/L groups (*n* = 3). The clustering showed that the replicates in each group clustered more within the group than with the other groups (**b**) top 100 differentially expressed genes (DEGs) across the control, 100 ng/L and 1 mg/L groups based on the DESeq2 output of 100 ng/L vs. control DE analysis (*p* < 0.05 and log_2_-fold change > 1 and transcript read count > 2000).

As shown in Figure 6a, the top 1000 genes present in the sequenced transcripts were compared between treatment groups, indicating that the 100 ng/L group clustered closer to the control than the 1 mg/L group. This appears to reflect the observed developmental patterns in the plants in which the 100 ng/L treatment group outwardly looked as healthy as the controls, unlike the stunted growth and leaf deformation observed in the 1 mg/L group. However, when the top 100 differentially expressed genes were compared, there was a closer clustering between the 100 ng/L and 1 mg/L groups (Figure 6b).

**Figure 7:**
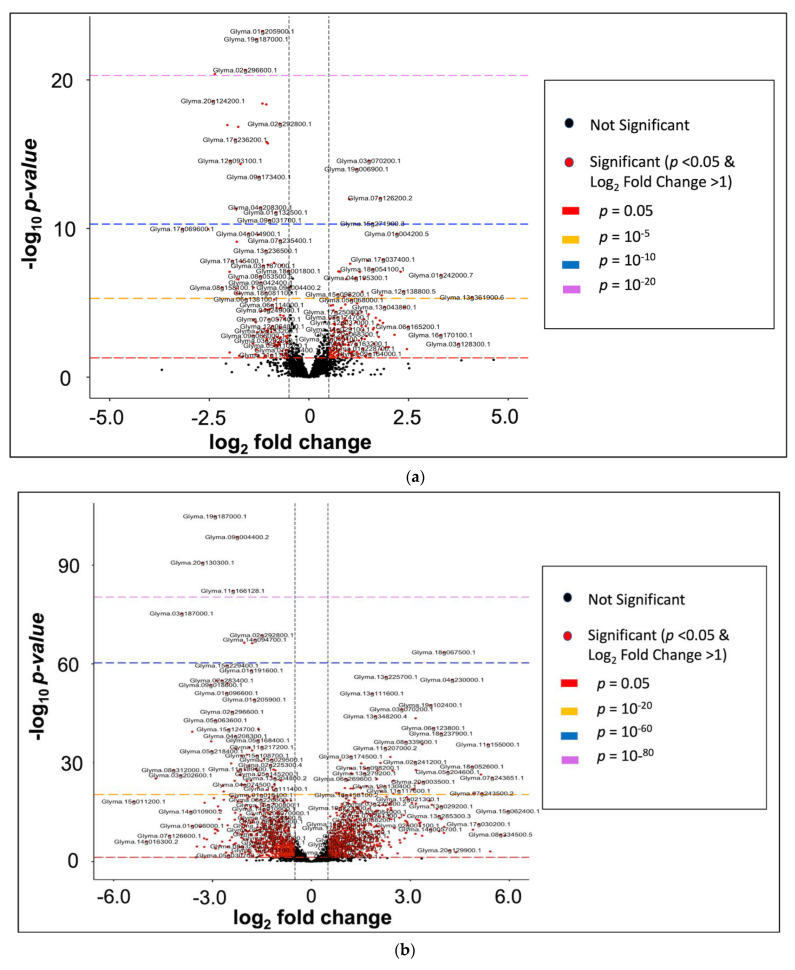
Enhanced Volcano plot of differentially expressed genes (DEGs) using gene transcript reads with counts greater than 2000 (**a**) in the 100 ng/L vs. control analysis. Note: Two genes (1 upregulated and 1 downregulated) fall outside the *x*-axis range. (See complete gene list in Supplemental S3—Sheet 1) and (**b**) in the 1 mg/L vs. control analysis. Note: Eight genes fall outside the *x*-axis range i.e., 2 upregulated and 6 downregulated (See complete gene list in Supplemental S3—Sheet 2). Red points indicate significant DEGs at *p* < 0.05 and log_2_-fold change > 1; log_2_-fold change < 0 = downregulated genes, log_2_-fold change > 0 = upregulated genes; Long dashes indicate different *p*-values thresholds.

The 100 ng/L group had 55 overexpressed genes and 55 suppressed genes having a log_2_-fold change > 1 at *p* < 0.05 (Figure 7a). The log_2_-fold change within this group clustered around −3.2 to 4. However, log_2_-fold change outliers of 25.6 (overexpressed) and −13.5 (suppressed) were observed for two genes, i.e., Glyma.05G033500 and Glyma.08G124600, respectively. Much of the 100 ng/L DEGs had *p*-values that clustered around 0.05 to 10^−10^. For the 1 mg/L group (Figure 7b), 395 genes were overexpressed while 358 genes were suppressed at *p* < 0.05 (log_2_-fold change > 1). The log_2_-fold change ranged from −10 to 8 for most of the gene transcripts with outliers of −25.7 and −29.2 for suppressed genes Glyma.10G172100 and Glyma.14G094700, respectively, and one overexpressed gene, Glyma.05G033500 (log_2_-fold change = 28.8). About 50% of the DEGs in the 1 mg/L group were below a *p*-value threshold of 10^−20^.

**Figure 8:**
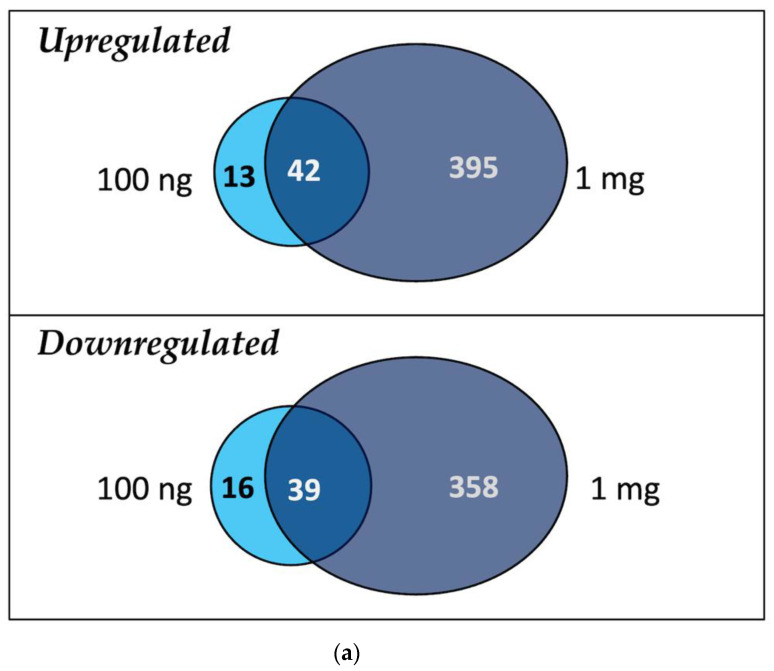

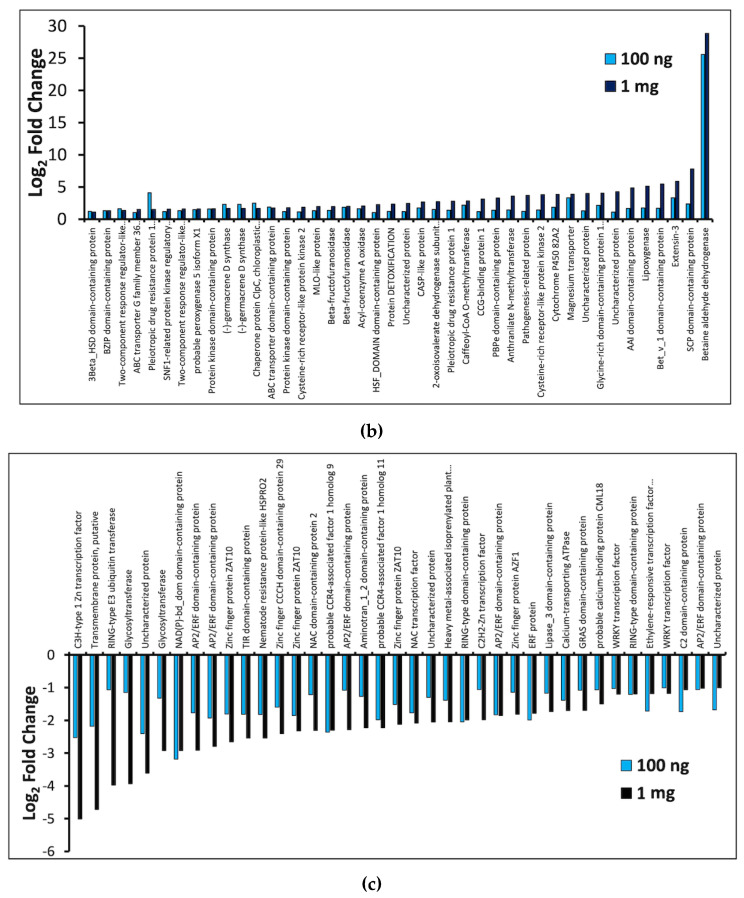
(**a**) Venn diagram showing the number of similar and different upregulated and downregulated genes between DEGs in the 100 ng/L vs. control and 1 mg/L vs. control analysis. Plots of log_2_-fold change against similar DEGs (intersection) in (**b**) upregulated and (**c**) downregulated DEG profiles in the 100 ng/L and 1 mg/L groups.

Comparing the DEG profiles in both groups, 73.6% of up- and downregulated genes in the 100 ng/L group were similarly expressed in the 1 mg/L group (Figure 8a). Indicators of response to a xenobiotic were evident in both treatment groups. Genes coding for stress response transporters and proteins such as pleiotropic drug resistance protein 1 isoform A, chaperone protein ClpC chloroplastic isoform A, lipoxygenase, SNF1-related protein kinase, protein DETOXIFICATION, pathogenesis-related protein, and isoforms of cytochrome P450 were overexpressed in both the 100 ng/L and 1 mg/L treatments. The gene that encodes betaine aldehyde dehydrogenase was the most overexpressed (Figure 8b).

Of the similarly downregulated genes, 18% are involved in the ethylene signaling pathway (e.g., ERF, NAC, WRKY; Figure 8c). For the suppressed genes, YTH domain-containing protein encoding gene and glycine cleavage system P protein gene were the most suppressed for the 100 ng/L and 1 mg/L groups, respectively (Supplemental S1—Figures S5 and S6).

**Figure 9:**
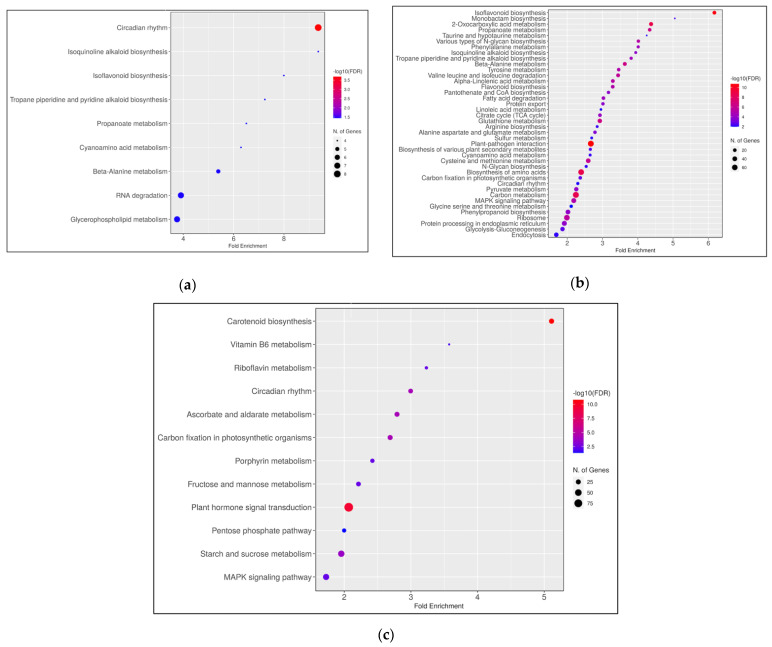
KEGG pathways and their Fold Enrichment at FDR < 0.05 using a list of the: (**a**) 100 ng/L group upregulated genes, (**b**) 1 mg/L group upregulated genes, and (**c**) the 1 mg/L group downregulated genes. The analysis was carried using DEGs at *p* < 0.1 and log_2_-fold change > 1. The Fold Enrichment was calculated as the percentage of differentially expressed genes for each group belonging to a pathway, divided by the corresponding percentage of genes in the background. FDR is calculated based on nominal *p*-value from the hypergeometric test [29]. (Note: Only top 40 pathways of 56 are displayed for the 1 mg/L upregulated gene list. For the complete list see Supplemental S4).

In order to discover gene networks stimulated and/or repressed by PFBA, KEGG pathway enrichment analysis was performed, following the recommendation of Hong et al. [28] to analyze up- and downregulated genes separately. The analysis was carried out using DEGs at *p* < 0.1 and log_2_-fold change > 1 (See gene list in Supplemental S3—Sheets 3 and 4). When looking at the KEGG analysis at a False Discovery Rate (FDR) < 0.05, we observed nine upregulated pathways in the 100 ng/L group, with none that were downregulated (Figure 9a). The Circadian Rhythm pathway was the most upregulated with fold enrichment of 9.4 in this group. For the 1 mg/L group, 56 and 12 pathways were up- and downregulated, respectively (Figure 9b,c). The isoflavonoid biosynthesis pathway was the most upregulated (Fold Enrichment = 6.2; FDR = 3.5 × 10^−11^). The carotenoid pathway showed the greatest Fold Enrichment i.e., 5.1 (FDR = 1.6 × 10^−11^) of all the downregulated pathways in the 1 mg/L group.

All of the upregulated pathways in the 100 ng/L group were also upregulated in the 1 mg/L group. We also found three pathways were both up-and downregulated in the 1 mg/L group i.e., Circadian Rhythm (FDR: up = 2.3, down = 3), Carbon Fixation (FDR: up = 2.4, down = 2.7), and MAPK signaling (FDR: up = 2.2, down = 1.7). Interestingly, the Circadian Rhythm pathway was the only differentially enriched intersecting pathway between both groups. We noticed that 8 and 14 genes involved in the Circadian Rhythm pathway were, respectively, upregulated in the 100 ng/L and 1 mg/L groups (Supplemental S1—Table S3a,b and Supplemental S4—Sheets 1 and 2) while 22 genes were downregulated in the 1 mg/L group (Supplemental S1—Table S3c and Supplemental S4—Sheet 3). Of the 14 upregulated KEGG genes in the 1 mg/L group, 4 encode for Chalcone synthase isoforms. Up- and downregulation of the similar clock genes was observed, which included a number of alternatively spliced genes. This may be a misregulation that is representative of plants undergoing severe stress in response to the high content of the toxic compound. We also observed that the Cryptochrome-1 and Cryptochrome-2 encoding genes were only upregulated in the 100 ng/L groups and not in the 1 mg/L group. By contrast, Cryptochrome-1 was downregulated in the 1 mg/L treatment.

## 3. Discussion

Previous studies have demonstrated PFBA transport from soils to the aerial parts of plants, [3,22] but it has not been fully understood how this exposure (especially at environmentally relevant levels) may affect critical physiological and developmental indices [1]. We report that using soybean (*Glycine max*) as our model system, PFBA is recognized as a xenobiotic by the plant as indicated by multiple stress response pathways that were stimulated upon exposure. However, only at high exposure concentrations is the chemical capable of exerting an observably toxic outcome, as at an environmentally relevant concentration of 100 ng/L plants showed a greater increase in height and chlorophyll *a* content than control plants.

### 3.1. PFBA and Plant Stress

PFBA induced significant oxidative stress even at the lowest treatment group of 100 pg/L (Figure 4a). Both SOD and CAT activities were also depressed in all treatment groups, consistent with studies showing inhibition of superoxidase dismutase (SOD) and catalase (CAT) activity [17,18,30,31]. Surprisingly, neither of these genes were downregulated in gene expression analysis, indicating that PFBA either leads to enhanced degradation of the enzymes after translation of the gene transcripts or is a direct inhibitor of these enzymes. It should be noted that long-chain PFAS have been observed to inactivate SOD activity, but only at considerably high levels [15,18,21,31]. The reduction of H_2_O_2_ (Figure 4b) is likely a direct result of lowered SOD activity. As stomatal opening is also linked to low H_2_O_2_ levels [32], this may contribute to the comparatively higher aerial accumulation of PFBA since PFAS uptake has been hypothesized to be enhanced by transpiration [3,33,34].

### 3.2. PFBA-Induced Toxicity and Stress Response Genes

At high concentration (1 mg/L of irrigation water) PFBA has a broadly toxic effect, as observable by alterations in plant physiology, i.e., leaf chlorophyll/morphology, and plant height. This is consistent with studies such as that by Lan et al. [35] in which exposure of wheat plants to 2 mg/kg of a PFBA/perfluorobutane sulfonic acid (PFBS) mixture in soil reduced the synthesis of Chl *a* by 34.9%. Other researchers [21] have also reported leaf chlorosis and decreased chlorophyll levels in lettuce after exposure to 20–200 µM PFOA for 2 weeks. Photosynthetic activity is one of the most critical factors influencing the growth rate of plants [36,37], and high concentrations of long-chain PFAS have been shown to downregulate genes involved in photosynthesis [22]. We also observed the downregulation of several genes involved in the generation of chlorophyll and carotenoids, such as chloroplast magnesium chelatase and phytoene synthase. The Calvin Cycle was also among the suppressed pathways using KEGG analysis.

The reduction of flavonoid content in the two highest treatment groups, particularly at 1 mg/L (Figure 2), points to enhanced UV damage as one potential mechanism for the deformed and discolored leaves observed in these treatment groups [38]. The upregulation of multiple genes in the synthesis and modification of flavonoids (Supplemental S3—Sheet 2 and also see [39]) may be a compensation effect. The mechanism for the loss of flavonoids during PFBA toxicity should be the focus of future studies.

The most upregulated gene in both groups encodes betaine aldehyde dehydrogenase (BADH; Figure 8b). The BADH enzyme catalyzes the production of glycine betaine, which is an essential osmotic protectant in plants in response to abiotic stressors such as drought and salinity [40,41]. The very high expression of this gene suggests that the plants utilized glycine betaine in a non-enzymatic stress-response pathway, as was observed with other PFAS studies such as non-enzymatic ROS quenching in lettuce [19,30]. The role of alkaloids as secondary antioxidant molecules in plants in response to abiotic stress is also well established [20,30,42,43,44,45]. Thus, the enriched isoquinoline alkaloid biosynthesis and tropane piperidine and pyridine biosynthesis pathways in both the 100 ng/L and 1 mg/L groups may also represent a non-enzymatic oxidative stress response.

Of interest was the downregulation of genes related to ethylene signaling. Eighteen percent of similarly downregulated genes in both treatment groups represented members of NAC, WRKY, and AP2/ERF transcription factor families involved in the ethylene signaling pathway [46,47]. As the accumulation of H_2_O_2_ is known to increase the expression of these transcription factors [47,48], the reduced H_2_O_2_ in all treatment groups is consistent with the observed downregulation. This suggests that soybean plants upregulate specific stress-response pathways to cope with a xenobiotic such as PFBA.

### 3.3. PFBA and Hormesis

Despite the obvious toxicity of the 1 mg/L treatment, plant height, leaf width, and Chl *a* content were all stimulated at lower concentrations, peaking at 100 ng/L (Figure 1 and Figure 2). Similar low-dose dose stimulation has been reported for long-chain PFAS [20,22,49,50]. Visual inspection of up- and downregulated genes in both groups (Figure 8b, c) did not reveal any candidate genes to explain what may have contributed to the observed PFBA-induced stimulation in this study. However, KEGG analysis demonstrated that the Circadian Rhythm pathway was the only differentially regulated pathway between both the 100 ng/L and 1 mg/L groups, with a significant enrichment in the 100 ng/L group (Fold enrichment > 9, FDR = 2 × 10^−4^).

Plants with optimized circadian clocks have adaptive advantages, increased chlorophyll content, enhanced photosynthetic capacity, higher biomass accumulation, optimized nighttime carbon utilization, and improved tolerance [51,52,53,54,55]. The circadian rhythm has been shown to be responsive to abiotic stress and to regulate downstream stress response genes, thus possibly conferring an adaptive advantage [56,57,58,59]. For example, exposure of soybean to alkaline stress lengthened the expression period of CIRCADIAN CLOCK ASSOCIATED1/LATE ELONGATED HYPOCOTYL (CCA1/LHY), while heat shock elongated the expressions of PSEUDO-RESPONSE REGULATOR 5 and 7 (PRR5 and PRR7) and FKF1 [60].

Of particular interest was the upregulation of Cryptochrome-1 (CRY1) and Cryptochrome-2 (CRY2) encoding genes in the 100 ng/L treatment group. The CRY proteins are photosensors that regulate growth and development while also serving as an entrainment for the circadian clock [61,62,63]. They also have been shown to hetero-oligomerize in order to carry out their function [64].

## 4. Materials and Methods

### 4.1. Chemicals, Standards, and Reagents

Hexafluoro butyric acid (PFBA; 98% purity), methanol (LC-MS grade), sulfuric acid, titanium tetrachloride, hydrogen peroxide (3%), potassium phosphate monobasic, and dibasic (for phosphate buffer preparation), quercetin and gallic acid were purchased from Sigma-Aldrich (Allentown, PA, USA). Bovine serum albumin (BSA), Coomassie reagent, 2′, 7′ -dichlorofluorescein diacetate (H_2_DCFDA), and SOD kit were purchased from Thermo Fisher Scientific (Allenstown, PA, USA). Nanopure water (Thermo Fisher Scientific) was used to prepare all solutions.

### 4.2. Pot Experiments

Soybean plants have been extensively used as models for PFAS uptake studies due to their important economic value [3,15,65]. Seeds were purchased from Jonny’s Selected Seeds (Winslow, ME, USA). They were surface sterilized, and two seeds were sown in polypropylene pots containing 80 g of wet sterilized soil (Pro-Mix BX Mycorrhizae) in a climate-controlled chamber (*day*: 15 h, 27 °C, 70%RH; *night*: 9 h, 23 °C, 70%RH). Each pot was placed in a pre-rinsed polypropylene bag to prevent water drainage which might lead to cross-pot contamination. Five treatment groups (4 pots per treatment) were designed, and irrigation was accordingly commenced with PFBA-spiked water at concentrations of 100 pg/L, 10 ng/L, 100 ng/L, 100 μg/L, and 1 mg/L, respectively. The experimental controls were irrigated with Nanopure water (Barnstead, Thermofisher). Equal volumes of water were used in irrigating all plant samples throughout the 5-week growing period i.e., 1.4 L per pot.

### 4.3. Physiological Endpoints Determination

Chlorophyll content and plant height were studied in the exposed and control plants to understand the impact of PFBA on essential physiological and developmental endpoints. Leaf chlorophyll content was measured on the soybean plant leaves at 5 weeks just before the plants were harvested, using a Chlorophyll Content Meter (Apogee, Logan, Utah). For each plant, the mean chlorophyll content was measured from representative leaves across the different height ranges of the plants. Plant height was measured just before harvesting.

### 4.4. Flavonoid Content Determination

At 5 weeks, representative leaf samples from all treatment groups and the control were harvested, flash-frozen in liquid nitrogen, ground to powder, weighed (100 mg), and then homogenized with 1 mL of 50:50% acetonitrile/water. The mixture was thereafter sonicated for 10 min at room temperature. The sonicated solution was centrifuged at 8000× *g* for 10 min and the extracts were collected into Eppendorf tubes. For the flavonoid content determination, the method described by Ghasemi et al. [66] was used. Briefly, 20 µL of plant extract, 112 µL of distilled water, 60 µL of 80% methanol, 4 µL of 0.5 M aluminum trichloride solution, and 4 µL of 1 M potassium acetate were added to wells in a 96-well plate. After incubating at room temperature in the dark for 30 min, absorbance measurement was taken at 415 nm. The total flavonoids are reported as mg quercetin equivalents (QE)/g FW.

### 4.5. Stress Enzymes and Metabolites Quantification

For the determination of critical stress metabolites and enzymes, enzymatic extracts from the soybean plants were prepared from leaf samples from each treatment group. Briefly, harvested leaf samples were flash-frozen and homogenized using liquid nitrogen, and 100 mg sample was further homogenized using 1 mL of pre-chilled 0.1 M phosphate buffer under ice-cold conditions. These extracts were further processed for the different assays.

#### 4.5.1. Total Reactive Oxygen Species

Enzymatic extracts were centrifuged at 6000× *g* for 20 min and 100 µL of the supernatant was transferred to a 96-well plate. The determination was carried out following a method described in [67]. To the extracts in the plate, 5 µL of 1 mM H_2_DCFDA was added. The plate was then incubated in the dark for 30 min and fluorescence measurement was carried out at excitation and emission wavelength sweeps of 460/40 and 528/20 nm, respectively.

#### 4.5.2. Superoxide Dismutase Assay

The SOD content was determined following the protocol described in the kit (Thermofisher Scientific, Waltham, MA, USA). Briefly, 10 µL enzymatic extracts used for the protein determination (diluted 1:1 with assay buffer) were transferred to a 96-well plate and 50 µL of the substrate was added. Thereafter 25 µL of xanthine oxidase was added to each well and the plate was incubated at room temperature for 20 min. Absorbance measurement was taken at 450 nm and the SOD concentration was calculated from a standard curve. Final Concentration data were expressed as Unit SOD per mg protein (U/mg protein).

#### 4.5.3. Hydrogen Peroxide Content

Determination of hydrogen peroxide content was carried out using a modification of the method described in [68]. Enzymatic extracts were centrifuged at 6000× *g* for 20 min and to 150 µL of the supernatant, 50 µL of 0.1% titanium tetrachloride in 20% *v*/*v* H_2_SO_4_ was added. The solution was centrifuged at 6000× *g* for 15 min and transferred to a 96-well plate where absorbance was determined at 415 nm. The hydrogen peroxide concentration was computed using 0.28 µmol^−1^ cm^−1^ as the extinction coefficient and expressed as µmol/g FW.

#### 4.5.4. Catalase Assay

Determination of the catalase (CAT) activity was carried out on the enzymatic extracts used for the protein assay. The quantitation method used is described in [68,69]. Briefly, 50 µL of the enzymatic extracts were transferred to a 96-well plate and 247 µL of phosphate buffer (pH 7) and 3 µL of 1 M H_2_O_2_ were added to the wells. Blank solutions containing only the phosphate buffer and hydrogen peroxide were used to correct for non-enzymatic degradation of hydrogen peroxide. The decreasing absorbance at 240 nm was followed over 3 min and the rate of concentration change was determined. The concentration was determined using 39.4 mM^−1^ cm^−1^ as extinction coefficient and expressed as mmol m^−1^/mg protein.

### 4.6. RNA Isolation and Sequencing

Isolation of RNA was carried out on triplicate samples from the Controls, 100 ng, and 1 mg treatment groups using the RNeasy Plant Mini Kit (Qiagen) following the recommended protocol. The quality and concentration of the isolated RNA were assessed using an Agarose Gel electrophoresis and a NanoDrop Spectrophotometer. mRNA preparation, quality check, and sequencing were carried out at a commercial lab facility (Psomagen, Rockville, MD, USA). Libraries were prepared using the TruSeq Stranded mRNA LT Sample Prep Kit (Illumina Inc, San Diego, CA, USA). The sequencing library was prepared by random fragmentation of the cDNA generated from the mRNA sample, followed by 5′ and 3′ adapter ligation. Prior to sequencing, clusters were generated by loading the library into a flow cell where fragments are captured on a lawn of surface-bound oligos complementary to the library adapters. Each fragment is then amplified into distinct, clonal clusters through bridge amplification. Sequencing was thereafter carried out on paired-end reads containing 150 base pairs using an Illumina HiSeq 2500 platform.

### 4.7. Transcriptome Analysis

The raw reads from the sequencer showed a range of 30,000,000 to 35,000,000 total paired reads from all samples. Processing of the reads was commenced by the removal of the sequencing adaptors using trimmomatic [70]. Bases with quality scores below 25 were also trimmed off and the quality of the reads was confirmed using FastQC (Babraham Bioinformatics, UK). An index to the soybean reference genome was built with the Fasta file from the Joint Genomic Institute (JGI) Phytozome database (*Gmax_508_v4.0.fa*) using hisat2-Build tool and the processed fast read files were mapped against the built index using hisat2 and SAMtools. Mapping alignment data showed a range of 80–89% alignment rate for all nine samples. The resulting sorted binary alignment map (BAM) files were processed with Stringtie and python scripts to generate raw gene transcript counts. The sequence data files generated from this research were deposited in the NCBI’s SRA database (Bioproject Ascension ID: PRJNA850028)

Prior to all gene expression analysis, low count reads were excluded by using only transcripts with greater than 2000 read counts. The average gene counts across all groups (control, 100 ng and 1 mg) were then used to generate a heatmap in a decreasing order using RStudio [71]. The top 1000 of these expressed genes was reported. For processing the differentially expressed genes (DEGs), RStudio [71] was also used. Differentially expressed genes between the 100 ng vs. control and between the 1 mg vs. control were determined and normalized for each gene using DESeq2 [72] within R. Significant DEGs were determined to be genes with log_2_-fold change greater than 1 with a *p*-value less than 0.05. For identification of the DEGs, their transcript ids were identified from *Glycine max* Annotation file (soybase_genome_annotation_v4.0) downloaded from Soybase [73]. The names and descriptions adopted for the genes are the topmost descriptive Uniref100 Viridaeplantae BLASTP Hit from the Uniref100 database.

### 4.8. Data Analysis

The 2016 Microsoft Excel Data Analysis and the R software packages were used for processing obtained data. The Shapiro–Wilk test for normality was used to evaluate the normality of the various experimental data prior to analysis. Analysis of variance of the chlorophyll content, plant height, and total protein content between the controls and each of the treatment groups was carried out. Obtained data were subsequently modeled using the R [71] with the add-on package drc [74]. The data were fitted using the Brain–Cousens 4-parameter hormesis model (BC.4) where the lower horizontal asymptote, *c*, is fixed at 0. The BC model as defined by Brain and Cousens [75] is expressed as:

(1) $$f\;\left(x,\;b,\;c,\;d,\;e,\;f\right)=c+\frac{d-c+fx}{1+\exp\left(b\left(\log\left(x\right)-\log\left(e\right)\right)\right)}$$

The DEGs of the 100 ng/L and 1 mg/L were visualized using the enhanced volcano package in the R-computing environment at *p* < 0.05 log_2_-fold change greater than 1. Enriched pathways using gene ontology (GO) terms was determined using KEGG [76] on the ShinyGO platform [29] using DEGs at *p* < 0.1 at log_2_-fold change >1.

## 5. Conclusions

It is generally assumed that short-chain PFAS may only disrupt normal functions in biological systems at significantly higher concentrations compared to long-chain PFAS, due to their lower bioaccumulation potential [1]. However, our data suggest that environmentally relevant PFBA is able to modulate important biochemical and metabolic pathways involved in chlorophyll synthesis/accumulation, with a downstream effect on development. Moreover, comparably high PFBA concentration similarly impacted critical pathways as does PFOA and PFOS. A number of alternative splicing events of clock genes were detected at this concentration, hinting at post-transcriptional misregulation under extreme stress as has been reported in other systems [57,77,78,79].

Low dose stimulation and high dose inhibition, known as hormesis, have been observed in organisms responding to other chemical and environmental stressors [80,81,82,83,84]. Based on the KEGG pathway analysis from this study, we do suggest upregulation of circadian rhythm genes as one candidate pathway to explain the observed phenomenon of hormesis under physiologically relevant PFBA exposure. Though the 100 ng/L treatment did result in what appears to be improved plant growth, stress responses were evident in all treatment groups. Therefore, our data support the theory, as postulated by other researchers [26,27,85], that hormesis represents an overcompensation response to induced stress.

## Data Availability

The sequence data files generated from this research are available in the National Center for Biotechnology Sequence Read Archive (SRA) database (Bioproject Ascension ID: PRJNA850028).

## Supplementary Materials

The following supporting information can be downloaded at: https://www.mdpi.com/article/10.3390/ijms23179934/s1. References [25,27,85,86,87,88] are cited in the Supplementary Materials.
