# Supplementary material for: Perfluorobutanoic Acid (PFBA) Induces a Non-Enzymatic Oxidative Stress Response in Soybean (Glycine max L. Merr.)
Source: Int J Mol Sci. 2022 Sep 1;23(17):9934. doi: 10.3390/ijms23179934 (PMC9456126; doi:10.3390/ijms23179934)
Supplement: Supplementary file 1 [file ijms-23-09934-s001.zip › ijms-1798237-Supplemental S1.pdf]

Perfluorobutanoic Acid (PFBA) Induces a Non-Enzymatic Stress Response in Soybean (*Glycine max* L. Merr.)

Eguono W. Omagamre [1], Yeganeh Mansourian [1], Diamond Liles [1], Tigist Tolosa [2], Simon A. Zebelo [2], and Joseph S. Pitula [1,*]

[1] Department of Natural Sciences, University of Maryland Eastern Shore, Princess Anne, MD 21853, USA
[2] Department of Agricultural and Food Sciences, University of Maryland Eastern Shore, Princess Anne, MD 21853 USA
* Correspondence: jspitula@umes.edu

Supplemental S1

Table S1. Model parameters from Brain-Cousens 4 parameter dose-response fitting of the soybean plant height data. The non-significant *p*-value of the *f* at $p < 0.05$ and the Confidence interval of the *f* parameter spanning the zero range suggests insignificant hormesis.

Parameters	Estimate	Std. Error	t-value	p-value	Confidence Interval	
					2.5%	97.5%
b:(Intercept)	3.36	6.56	0.51	0.699	-80.00	86.71
d:(Intercept)	50.64	8.96	5.65	0.111	-63.17	164.44
e:(Intercept)	11.10	93.02	0.12	0.924	-1170.82	1193.03
f:(Intercept)	14.16	13.38	1.06	0.482	-155.84	184.15

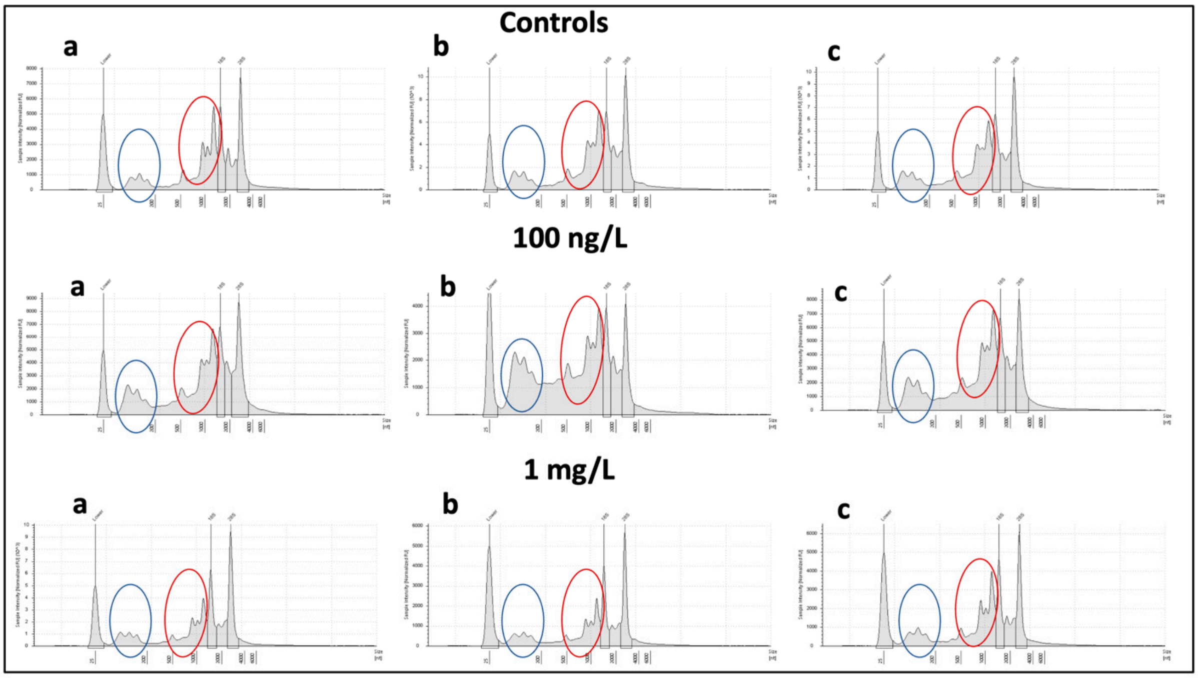

Figure S1. Ribosomal RNA bands were analyzed in total RNA extracts from triplicate soybean leaf samples from the Controls, 100 ng/L and the 1mg/L groups on an Agilent BioAnalyzer. The highlighted portions of the bands indicate areas that likely correspond to chloroplast ribosomes [25]. The peak areas in this region are largest in the 100 ng/L group that showed leaf chlorophyll stimulation. The 1mg/L group showed the least area around this chloroplast region.

Table S2. Model parameters from Brain-Cousens 4 parameter dose-response fitting of the soybean leaf chlorophyll data. The significant p-value of the f parameter at $p < 0.05$ suggests that the 19.93 f is significant hormesis.

Parameters	Estimate	Std. Error	t-value	p-value	Confidence Interval	
					2.5%	97.5%
b:(Intercept)	3.56	0.51	6.94	0.020	1.35	5.76
d:(Intercept)	232.81	2.99	77.76	0.000	219.93	245.70
e:(Intercept)	6.30	0.19	32.36	0.000	5.47	7.14
f:(Intercept)	19.93	3.40	5.86	0.028	5.30	34.55

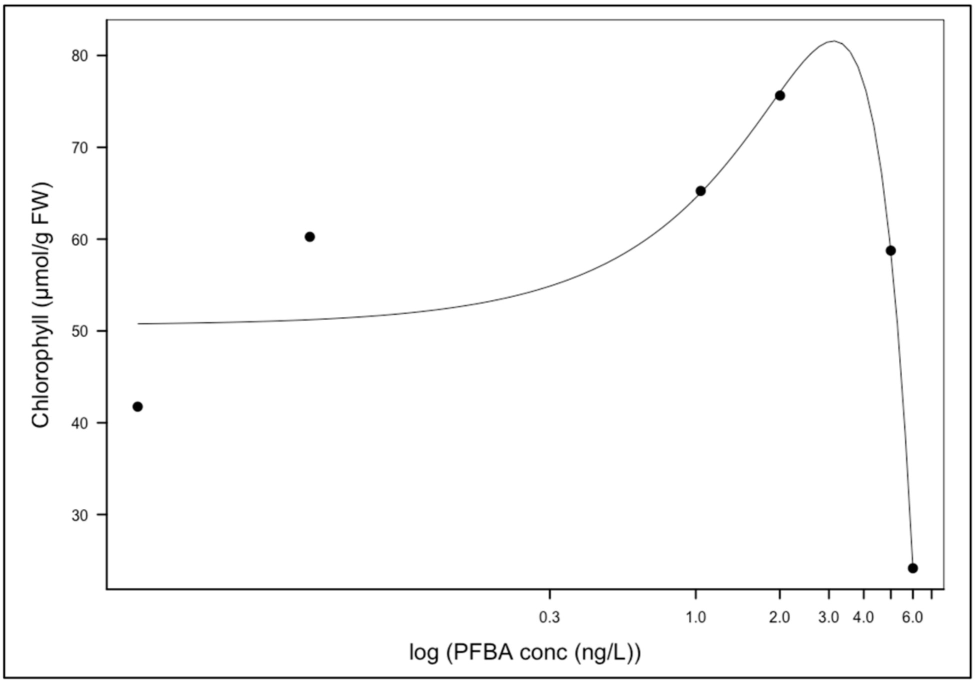

Figure S2. Curve fitting of the chlorophyll content against the treatment concentration. The asymmetric shape of the curve observed is consistent with the shape of non-monotonic curves that have been reported by other researchers in plant systems [27,86]. The hormetic dose zone (LDS/EC_{110}) and *dist*2 (M/LDS) were observed to be 1531-fold and 100-fold respectively. The hormetic dose zone and the *dist*2 are wider than the generalized characteristics observed in systems showing hormesis (80% of reported hormesis) [86]. However, 7% and 6% of all experiments showing hormesis, which were mostly in plant systems, have respectively reported hormetic dose zone and *dist*2 higher than 1000-fold and 100-fold [73,87,88].

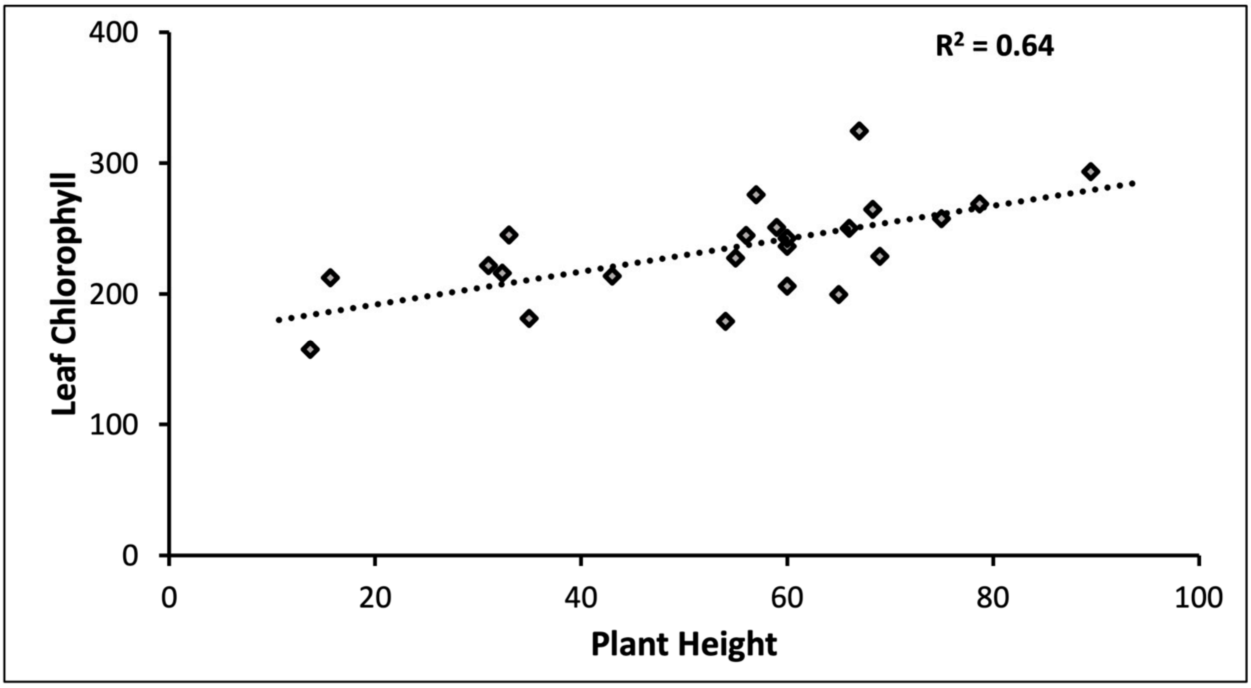

Figure S3: Correlation between soybean leaf chlorophyll content and plant height determined after 5 weeks of plant exposure (n = 24) with an r^2 of 0.64. The controls and all PFBA treatment groups were used as data points for the plot.

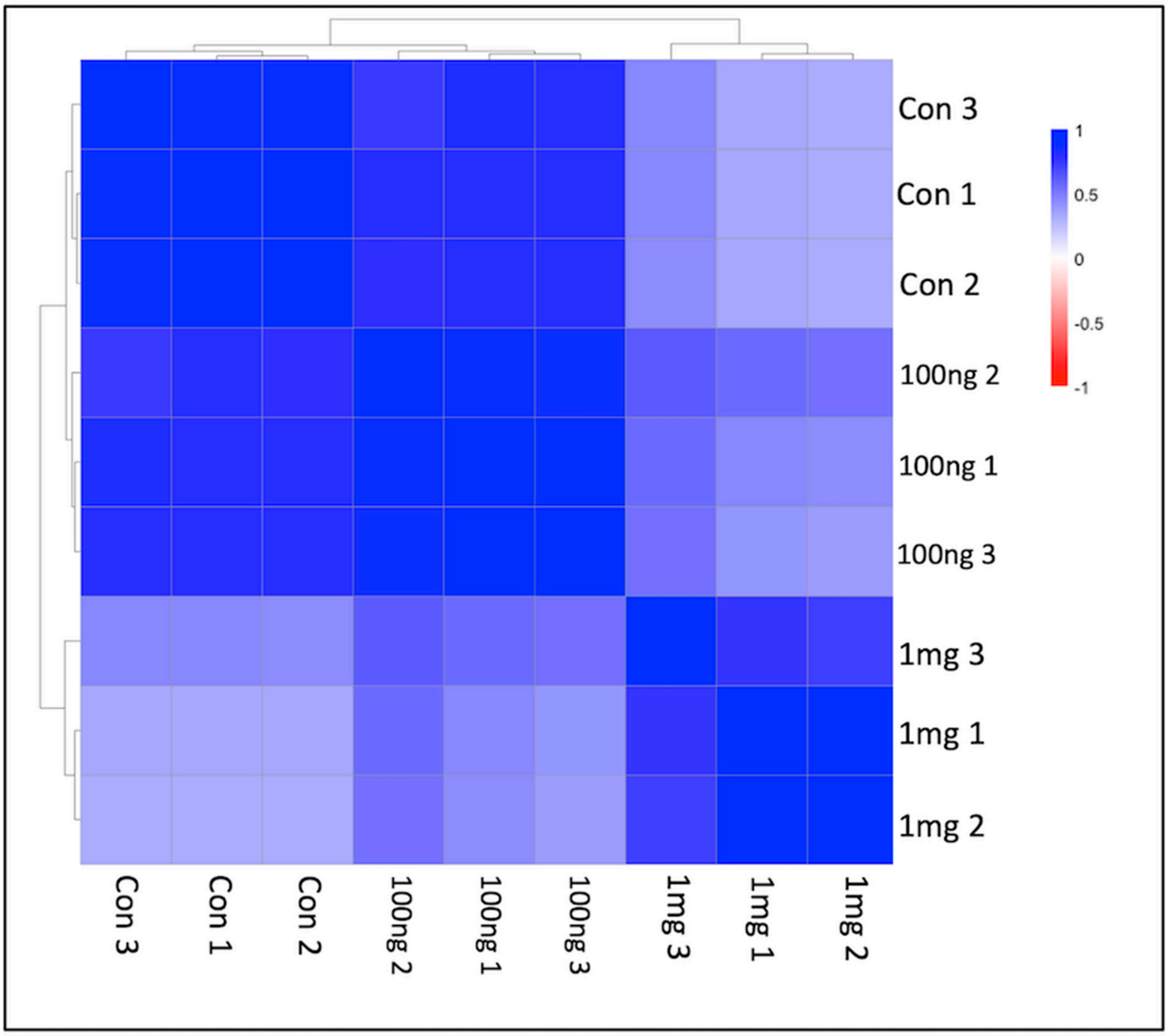

Figure S4: Heat map of total expressed genes showing cluster within and across treatment groups. The profile suggests all replicates within each treatments clustered together. The 100 ng/L group clustered closer to the control than the 1mg/L group which appeared reflective of the outward physiology of the plants.

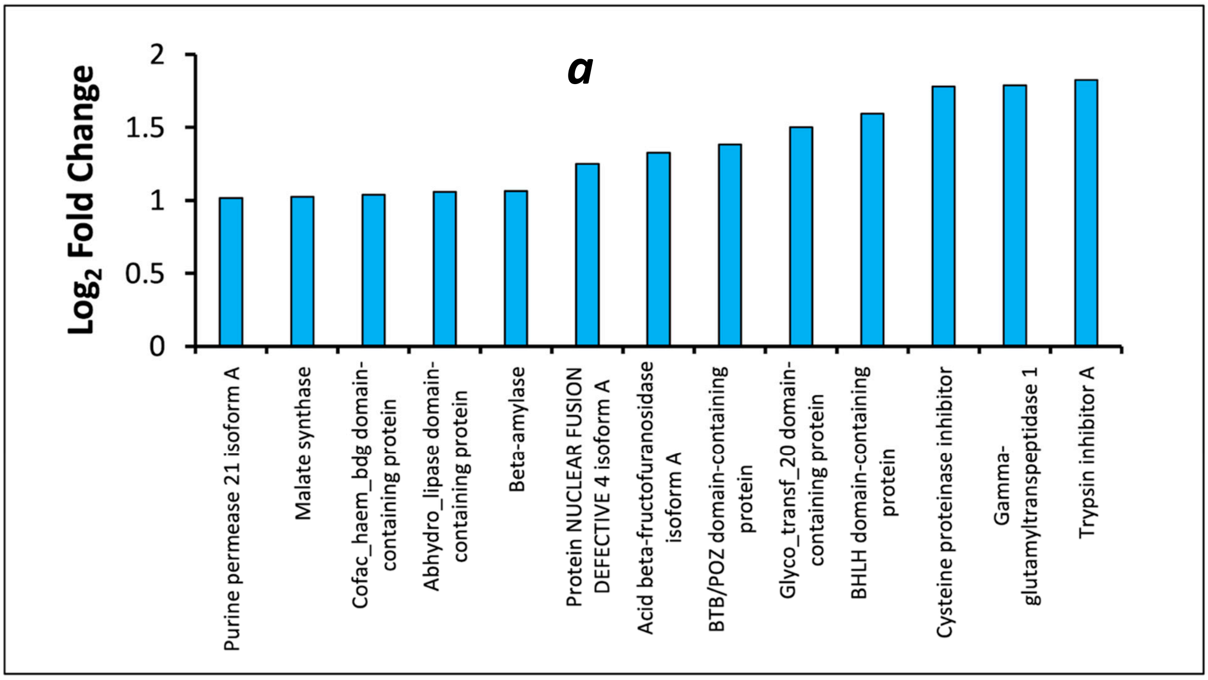

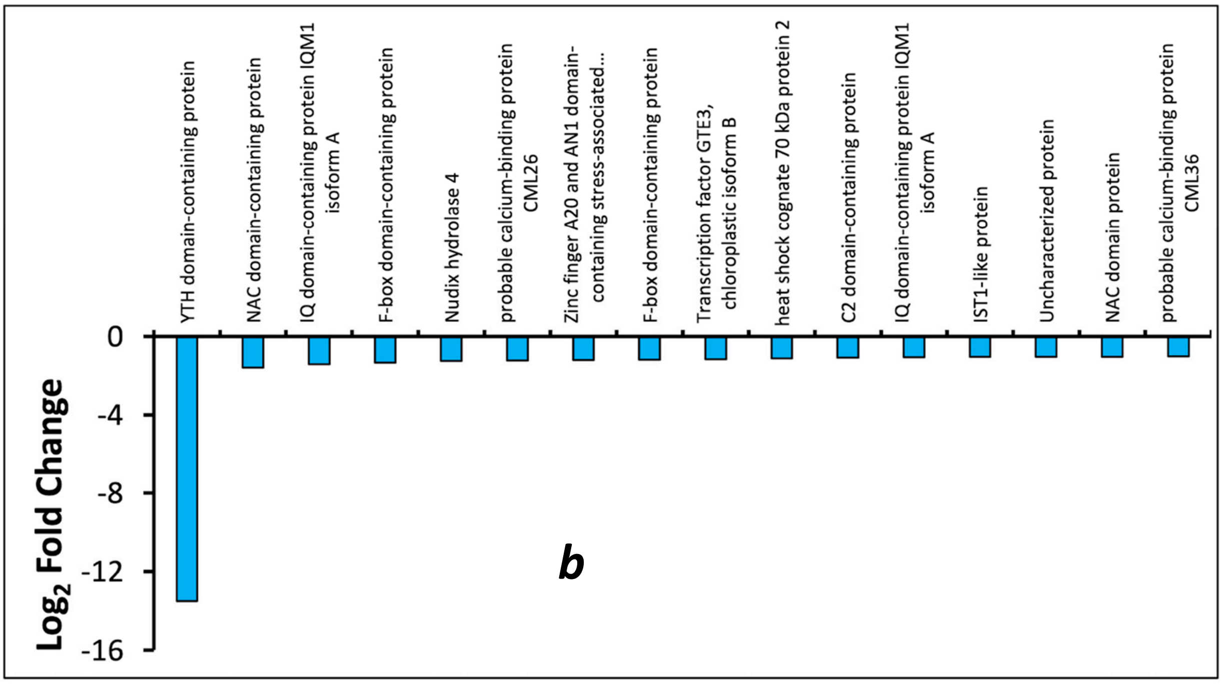

Figure S5: Plots of $\log_2$fold change against select (***a***) upregulated and (***b***) downregulated DEGs unique to the 100 ng/L group; at $p < 0.05$

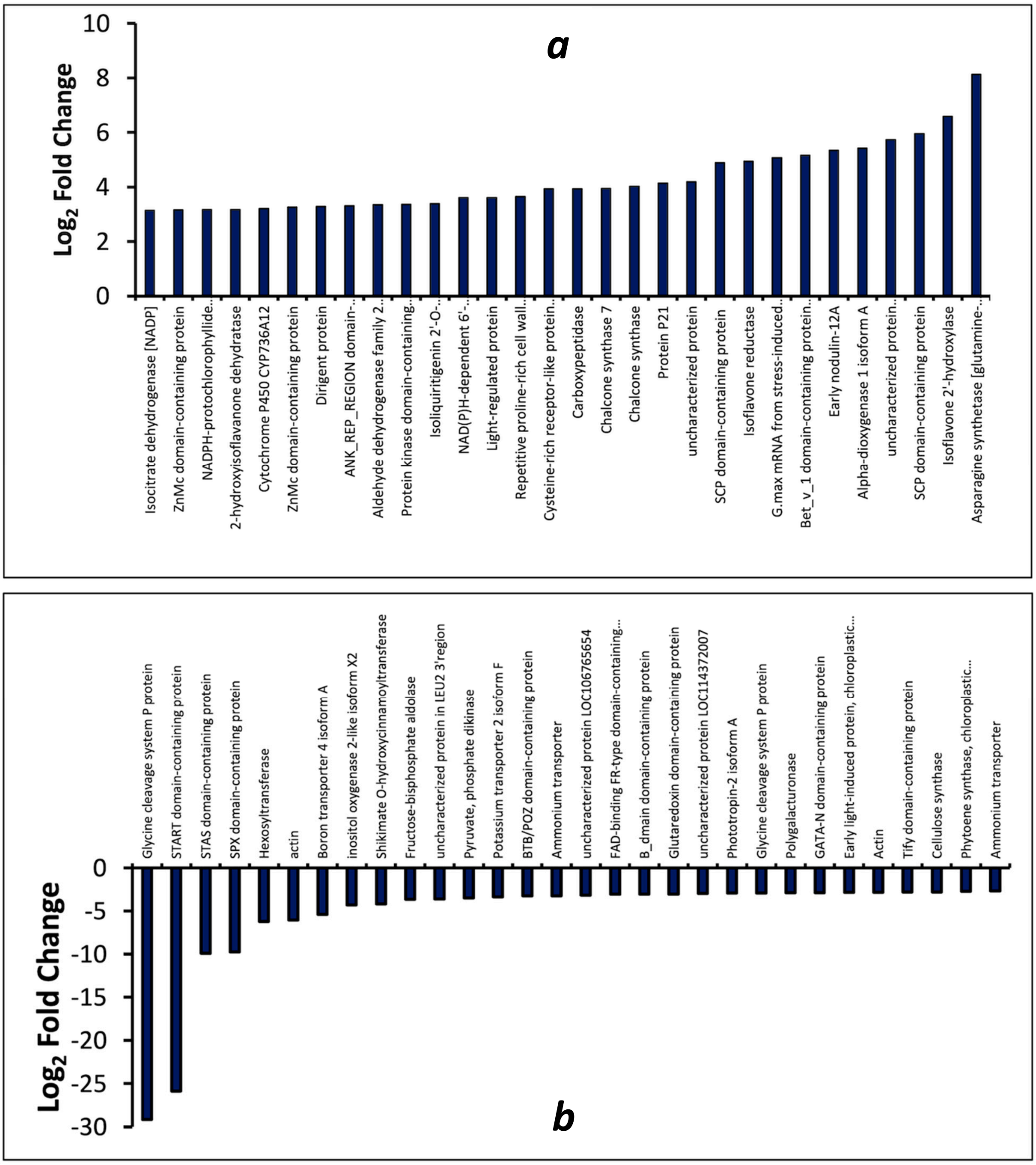

Figure S6: Plots of log₂fold change against select (***a***) upregulated and (***b***) downregulated DEGs unique to the 1 mg/L group; at $p < 0.05$. Please see Supplemental S3 for complete list

Table S3. List of Circadian Rhythm pathway genes highlighted by the KEGG analysis in the: (***a***) Upregulated gene cluster in the 100 ng/L group, (***b***) Upregulated gene cluster in the 1 mg/L group and (***c***) Downregulated gene cluster in the 1 mg/L group

Gene ID	Gene Name	Version Number of Gene Product (Log_2Fold Change)
Glyma.05g239400	Circadian clock-associated FKF1	1(1.3)
Glyma.06g103200	Cryptochrome-1	3(1.1)
Glyma.06g136600	Two-component response regulator-like PRR95 isoform A	1(1.4)
Glyma.07g048500	HTH myb-type domain-containing protein	15(1.9)
Glyma.08g197500	ELF3-like protein 2 isoform A	1(1.5)
Glyma.10g180600	Cryptochrome-2	1(2.6)
Glyma.19g224200	Phytochrome	6(23.2)
Glyma.20g170000	Gigantea-like protein 1	11(9.0)

(**a**)

Gene ID	Gene Name	Version Number of Gene Product (Log_2Fold Change)
Glyma.02g267800	E3 ubiquitin-protein ligase COP1	2(-5.1), 1(-1.6),
Glyma.16g017400	HTH myb-type domain-containing protein	5(-4.7), 18(-3.5), 13(-2.1), 10(-2.1), 1(-1.2)
Glyma.03g261300	Two-component response regulator-like PRR95 isoform B	1(-2.7), 4(-1.7),
Glyma.07g013500	ELF3-like protein 2 isoform X1	2(-1.8), 1(-1.6)
Glyma.19g224200	Phytochrome	7(-8.7),
Glyma.13g089200	Cryptochrome-1 isoform A	2(-1.2),
Glyma.19g260900	HTH myb-type domain-containing protein	8(-2.2), 3(-1.9)
Glyma.16g027200	Protein SUPPRESSOR OF PHYA-105 1 isoform A	1(-2.4)
Glyma.07g049400	Two-component response regulator-like PRR95 isoform A	16(-2.9)
Glyma.04g101500	Cryptochrome-1	2(-2.0)
Glyma.19g105100	Chalcone synthase	1(-2.4)
Glyma.05g214900	Protein SPA1-RELATED 2 isoform A	1(-1.2)
Glyma.10g221500	Gigantea	1(-9.7), 9(-1.9)
Glyma.16g092700	Transcription factor HY5-like	2(-6.7), 1(-3.0)
Glyma.20g091200	BHLH transcription factor	1(-1.7)
Glyma.14g049700	E3 ubiquitin-protein ligase COP1	4(-1.6)
Glyma.19g260400	Two-component response regulator-like PRR95 isoform F	4(-1.4), 1(-1.4)
Glyma.07g048500	HTH myb-type domain-containing protein	14(-2.4), 12(-2.4), 8(-2.1)
Glyma.03g261800	Late elongated hypocotyl and circadian clock associated-1-like protein 2	4(-2.4)
Glyma.16g018000	two-component response regulator-like PRR95 isoform X1	2(-2.5)
Glyma.03g225000	BHLH domain-containing protein	1(-2.4)
Glyma.14g174200	Cryptochrome-1	2(-1.9)

(**b**)

Gene ID	Gene Name	Version Number of Gene Product (Log_2Fold Change)
Glyma.01g228700	Chalcone synthase 7	1(3.6)
Glyma.04g050200	ELF3 protein	6(1.6)
Glyma.05g239400	Circadian clock-associated FKF1	1(1.6)
Glyma.06g136600	Two-component response regulator-like PRR95 isoform A	1(1.3)
Glyma.07g048500	HTH myb-type domain-containing protein	13(2.2)
Glyma.08g046500	PAS domain-containing protein	1(2.4)
Glyma.08g109200	Chalcone synthase 5	1(1.5)
Glyma.08g109500	Chalcone synthase 9	1(1.4)
Glyma.10g221500	Gigantea	12(3.5)
Glyma.11g011500	Chalcone synthase	1(3.7)
Glyma.17g231600	Protein EARLY FLOWERING 3	1(1.0)
Glyma.19g224200	Phytochrome	6(22.3)
Glyma.20g170000	Gigantea-like protein 1	11(7.1)

(**c**)
